# The succession of epiphytic microalgae conditions fungal community composition: how chytrids respond to blooms of dinoflagellates

**DOI:** 10.1038/s43705-023-00304-x

**Published:** 2023-09-26

**Authors:** Alan Denis Fernández-Valero, Albert Reñé, Natàlia Timoneda, Núria Pou-Solà, Jordina Gordi, Nagore Sampedro, Esther Garcés

**Affiliations:** Departament de Biologia Marina i Oceanografia, Institut de Ciències del Mar (CSIC), Pg. Marítim de la Barceloneta, 37-49, 08003 Barcelona, Catalonia Spain

**Keywords:** Microbial ecology, Microbial biooceanography, Biofilms, Fungal ecology, Metagenomics

## Abstract

This study aims to investigate the temporal dynamics of the epiphytic protist community on macroalgae, during the summer months, with a specific focus on fungi, and the interactions between zoosporic chytrid parasites and the proliferation of the dinoflagellates. We employed a combination of environmental sequencing techniques, incubation of natural samples, isolation of target organisms and laboratory experiments. Metabarcoding sequencing revealed changes in the dominant members of the epiphytic fungal community. Initially, fungi comprised < 1% of the protist community, mostly accounted for by Basidiomycota and Ascomycota, but with the emergence of Chytridiomycota during the mature phase of the biofilm, the fungal contribution increased to almost 30%. Chytridiomycota became dominant in parallel with an increase in the relative abundance of dinoflagellates in the community. Microscopy observations showed a general presence of chytrids following the peak proliferation of the dinoflagellate *Ostreopsis* sp., with the parasite, *D. arenysensis* as the dominant chytrid. The maximum infection prevalence was 2% indicating host-parasite coexistence. To further understand the *in-situ* prevalence of chytrids, we characterised the dynamics of the host abundance and prevalence of chytrids through co-culture. These laboratory experiments revealed intraspecific variability of *D. arenysensis* in its interaction with *Ostreopsis*, exhibiting a range from stable coexistence to the near-extinction of the host population. Moreover, while chytrids preferentially parasitized dinoflagellate cells, one of the strains examined displayed the ability to utilize pollen as a resource to maintain its viability, thus illustrating a facultative parasitic lifestyle. Our findings not only enrich our understanding of the diversity, ecology, and progression of epiphytic microalgal and fungal communities on Mediterranean coastal macroalgae, but they also shed light on the presence of zoosporic parasites in less-explored benthic habitats.

## Introduction

Fungi are an important component of the microeukaryotic community in aquatic environments. They engage in a wide variety of parasitic and saprophytic interactions [1] involving diverse members of the microbial community. Studies conducted in freshwater habitats have shown that fungal interactions range from competitive strategies for resource acquisition to the development of coexistence mechanisms based on niche differentiation [2]. While abiotic factors exert an impact on fungal community [3, 4], biotic factors, especially the influence of other organisms within the microbial community, also play a significant role [5]. Within the microbial community, the temporal patterns of fungal species composition range from seasonal and repetitive cycles to brief peaks in abundance. For instance, sudden increases in the abundance of zoosporic fungi belonging to the Chytridiomycota (referred to as chytrids) occur nearly simultaneously with diatom blooms [2, 6]. In addition, infections by members of the Chytridiomycota during the proliferations of other microalgal groups, including cyanobacteria [7, 8] and dinoflagellates [9, 10], have also been documented.

Fungi have been identified in diverse niches across the marine environment [11], including the benthic habitat [12]. The identification of a large number of fungal sequences in the benthic habitat indicates its role as a reservoir of zoosporic parasites, including chytrids, thus contributing to the overall diversity of the marine environment. In a previous study, the diversity of benthic microbial communities and the prevalence of specific fungal groups have revealed gradients spanning coastal, offshore, and deep-sea regions [13]. The authors observed that Chytridiomycota were dominant in the coastal zone, whereas Ascomycota and Basidiomycota were more prevalent on the seafloor. Another study specifically conducted within coastal area highlighted higher diversity of marine chytrid in sediment compared to the water column [9].

The characteristics of the benthic habitat, including inorganic substrate such as rocky surfaces, and organic substrates such as macroalgae and seagrass surfaces, exert significant influence on shaping the microbial community that develops into a biofilm. Notably, the organic substrates within the benthic habitat, primarily consisting of macroalgae and seagrass surfaces, create a nutrient-rich milieu abundant inorganic carbon. This, in turn, facilitates microbial colonization and promoting biofilm formation on available surfaces [14, 15]. The community composition of these epiphytic microorganisms is shaped by multiple biotic interactions that reflects the dynamic and complex interplay between the epiphytic community and the substrate [16, 17]. In the case of epiphytic fungi, these interactions involve colonization of the substrate through zoospores or hyphae, leading to the establishment of parasites, saprophytes, mutualists or endosymbionts [18]. An example of these interactions was observed in the study of epibiotic fungi on seaweed in the Red Sea, Egypt, wherein different available host seaweed species exerted strong selective pressure and temporal changes on the associated fungal populations [19]. Specific interaction among epiphytic species modulate the community composition on macroalgae. As an illustration, the fungus *Aspergillus versicolor*, found in the brown seaweed *Sargassum thunbergii*, generates compounds with antibacterial properties, thereby selectively limiting the growth of prokaryotes [20] Furthermore, the epibiotic fungus *Trichoderma virens*, isolated from the red alga *Gracilaria vermiculophylla*, has been reported to secrete compounds that inhibit the growth of phytoplankton species such as *Chattonella marina*, *Heterosigma akashiwo*, *Karlodinium veneficum* and *Prorocentrum donghaiense*, while also displaying toxicity towards certain zooplankton species [21].

In our study system, the colonization of macroalgae by epiphytic protist is a well-established process [22, 23]. The initial stages of epiphytic assemblage development on Mediterranean macroalgae are typically characterized by the presence of microalgae, particularly diatoms of the *Navicula*, *Nitzschia*, and *Coscinodiscus* genera. These diatoms possess the ability to attach to both inorganic or organic substrates and withstand hydrodynamic stress [24]. Moreover, certain benthic diatom species such as *Navicula* sp. and *Proschkinia* sp. have a negative impact on the growth of other microalgae, including the dinoflagellate *Ostreopsis*. As a result, they influence the composition of the epiphytic protist assemblage [25]. In the later stages of epiphyte biofilm formation on macroalgae, dinoflagellates of the genera *Gambierdiscus*, *Coolia*, *Prorocentrum*, *Amphidinium* and the aforementioned *Ostreopsis* become dominant.

Despite having knowledge of species succession within benthic epiphytic assemblages, the succession patterns of other heterotrophic protist groups, such as fungi, remain incompletely described and understood. Similarly, the temporal dynamics and interaction between chytrids and epiphytic dinoflagellates have yet to be investigated. We hypothesized that temporal changes in the composition of the epiphytic assemblage influence the presence and abundance of co-occurring fungi. Therefore, this study combines field sampling and laboratory experiments to investigate into the temporal succession of the epiphytic assemblage. Moreover, it explores the ecological interaction between Chytridiomycota and dinoflagellates over time, focusing on the dynamics of the most abundant chytrid, *Dinomyces arenysensis*, during its infection of its host, the bloom-forming dinoflagellate *Ostreopsis* sp.

## Materials and Methods

### Field sampling

Twelve weekly samplings were conducted from June to September 2021 at one station in the coastal locality of Sant Andreu de Llavaneres, NW Mediterranean Sea (41°33'07.5“N; 2°29'31.6“E) to determine the temporal dynamics of the epiphytic assemblage on macroalgae. The collection and treatment of the samples were based on the standard protocol for studying epiphytes associated with macrophytic substrates [26]. The macroalgal growing season extends from early June to late August [27]. At each sampling, samples of macroalgae were collected from the rocky surface of the study area at depths of 1–1.5 m. The sampled macroalgae represent the predominant species in the study area: *Halopteris scoparia*, *Jania rubens*, *Padina pavonica*, *Dictyota dichotoma* and *Cladophora* sp., and reductions in macroalgal cover or changes in epiphyte composition were not observed throughout the sampling period. The samples were placed in flasks containing 1 L of autoclaved seawater, which was used to wash and resuspend the epiphytic protist community. The macroalgae were then separated from the epiphytic resuspension and the fresh weight of the macroalgae (gFW) was determined. The resuspended samples were vigorously shaken until all biofilm was disintegrated. The epiphytic resuspensions were filtered through a 100 μm mesh and a 150 mL aliquot was fixed with Lugol’s iodine to determine the cell abundance of diatoms and dinoflagellates, by settling 10 mL of each fixed sample in Utermöhl chambers for 24 h [28]. Sedgewick-Rafter counting chambers were used when the cell abundance of certain species was too high. Cells were counted using a Leica-Leitz DMIRB inverted microscope equipped with a Jenoptik ProgRes C10 Plus camera. The dimensions of ten cells of each taxon were measured using the ProgRes CapturePro v2.8.8 software. Biovolumes were calculated using the appropriate geometric formulae [29], and measurements were taken for 15 cells per taxon. The results were expressed as cubic millimetres per gram of fresh weight of macroalgae (mm^3^ gFW^–1^).

### Metabarcoding sequencing and analyses

Each water sample derived from resuspension of the macroalgae was immediately filtered in two replicates using non-cytotoxic Nalgene™ polysulfone bottles and 8 μm polycarbonate filters with a diameter of 47 mm. The organisms on the filters represented the epiphytic assemblage spanning the 8 to 100 μm size fraction. The filters were stored frozen at −70 °C until processing. Genomic DNA from the filtered samples was extracted using the DNeasy PowerSoil kit (Qiagen) according to the manufacturer’s instructions, Metabarcoding sequencing of the V4 region of the 18S rDNA was performed using the universal primers EK-565F [30] and EUK-1134R-UnonMet (biased against metazoans [31]) to determine the protist community, defined as all eukaryotic microorganisms, including fungi, ranging from 8 μm to 100 μm. The 8 µm filter would exclude fungal zoospores and consequently result in an underestimation of fungal proportions. The fungal-specific primers ITS3-KYO2 [32] and ITS4 [33] were used to amplify the 5.8S and ITS2 fragments of the rDNA operon (hereinafter referred to ITS region). This region represents the best option for amplification and taxonomic identification of fungi [34]. Both the V4 18S rDNA and the ITS regions were sequenced on an Illumina PE300 system. The raw reads were trimmed from the adaptors using CUTADAPT [35]. The DADA2 pipeline [36] was used to filter and dereplicate the reads, trim low-quality sequences, merge paired-ends, and remove chimeras. The parameters maxEE = 4, 6 and truncLen = 270, 250 were applied to both regions (V4 18S rDNA and ITS), and minOverlap = 20 for V4 18S rDNA and minOverlap = 50 for ITS. The obtained ASVs were taxonomically assigned using a simple non-Bayesian taxonomy classifier, VSEARCH v2.8.1 [37], with the PR2 database version 4.12 [38] customized with new in-house sequences for the V4 region and the full UNITE + INSD dataset from UNITE community v.8.3 for the ITS segment [39] and with bootstrap cut-off = 0.6. Unassigned ITS sequences that could not be classified as belonging to fungal organisms were removed from the dataset and were not considered for the fungal community analysis. The amplicons were 490 bp for V4 18S rDNA and 450 bp for ITS. The data were normalized according to the relative abundance of ASVs per sample. Raw metagenomic reads were submitted to the NCBI Sequence Read Archive under Bioproject numbers PRJNA891539 and PRJNA891506, corresponding to the V4 18S rDNA and ITS region, respectively. The ASV tables, including the FASTA sequences and taxonomic classifications, are available in Supplementary File 1. Metabarcoding data were analysed using the package Phyloseq and all graphs were generated using the package ggplot2 from Rcran [40–42]. Nonmetric multidimensional scaling (NMDS) using Bray-Curtis distances was then performed to analyse the dissimilarity between samples and permutational multivariate analysis of variance using distance matrices (ADONIS) was performed to assess differences between sample groups.

### Dinoflagellate and chytrid sporangium cell counting by epifluorescence microscopy

Chytrid presence, defined herein as the detection of *Ostreopsis* cells infected with *Dinomyces*, was assessed using the filters representing the temporal samplings at the coastal locality. Infections were attributed to *Dinomyces* based on previously reported metabarcoding results, isolation of the strains, the identification of single cells, and the morphological identification of sporangia from laboratory cultures of *D. arenysensis*. A 10 mL fraction of the sample derived from the macroalgal resuspension was fixed with 10% formaldehyde (v:v) for 24 h, filtered through 10 μm polycarbonate filters, and stored at −70 °C. The filters were covered with a 0.1% agarose permeable film to prevent cell loss during further manipulation. The filters were then cut into triangular sections and placed on slides with a drop of DAPI oil to stain the DNA strands. The oil solution was prepared from 1.25 μL of 0.5 mg DAPI (4′,6-diamidine-2′-phenylindole dichlorohydrochloride) mL^−^^1^ and 125 μL of a pH 9.5 solution composed of 100 mL of glycerol, 2 mL of Vectashield, and 1 mL PBS. Stained filter sections were observed at 1000× magnification using an Olympus Bx61 epifluorescence microscope connected to an Olympus DP72 camera (Olympus America Inc.). The following fluorescence filters were used for DAPI detection: excitation BP330-385, dichroic mirror DM400, and barrier filter BA420. The infection prevalence (%) was calculated as the percentage of infected *Ostreopsis* cells with respect to the total number of cells. The determination of infection prevalence in natural samples was based solely on the presence of mature sporangia, visualized using epifluorescence microscopy.

### Isolation, culture of chytrids, and identification of cultured strains by single-cell PCR

All cultured strains used in this study were obtained from the stock culture collection of the Institut de Ciències del Mar (ICM-CSIC). In the laboratory, chytrid cultures were established by transferring 5 mL aliquots of the resuspended epiphytic assemblages to 6-well plates for incubation. For samples with *Ostreopsis* abundances < 10^3^ cells g FW^−^^1^, healthy cells from an *Ostreopsis* sp. culture (strain ICMB293) were added to the incubations to increase host density and promote parasite infection. All incubations were maintained in chambers at 20 ± 1 °C and subjected to a 12:12 h photoperiod (light:dark). Illumination was provided by LED fluorescent tubes with a photon irradiance of ~40 μmol photons m^−^^2^ s^−^^1^. The incubations were periodically checked for the presence of chytrid infections for up to 2 weeks. Infected dinoflagellate cells were manually isolated to establish clonal chytrid strains. The chytrids were grown in 24-well plates by transferring a 1 mL aliquot of mature sporangia every 3–4 days to a well containing 1 mL of an exponentially growing uninfected host stock culture. An *Ostreopsis* sp. strain (ICMB293) maintained under non-axenic conditions in F/2 medium and a salinity of 36 was used to maintain the chytrid strains. Samples from the chytrid cultures were collected and stained with Alcian blue (1% solution) to identify the polysaccharide structures constituting the mucilage of the host. The isolated strains were observed using a Leica DMIRB inverted microscope. Photographs were taken at 40–60× magnification using a Jenoptik ProgRes C10 Plus camera.

Individual mature sporangia were isolated from the parasitoid strains using glass micropipettes and then subjected to further molecular characterization. The single-cell protocol was implemented following [43] and [44]. A first PCR was performed using the primers EK82F and 28S-1611R [45, 46], to amplify the entire ribosomal operon. The PCR product served as a template to amplify the partial V4 18S rDNA gene, using the specific primers CRYPTO2-2F and EUK-1520R [47, 48]. Four μL of each product was electrophoresed on a 1.2% agarose gel. The gel was stained with SYBR Safe (Invitrogen) to visualize the bands and confirm the correct amplicon length. For samples with poorly visualizable bands, a third semi-nested PCR was performed using the specific primers CRYPTO2-2F and AU4v2 [48] and the product of the previous PCR as template. Finally, Sanger sequencing with forward and reverse primers was performed by an external service (Genoscreen, Lille, France).

### Infection dynamics

The temporal dynamics of *Dinomyces arenysensis* infection were examined using strains isolated during *Ostreopsis* proliferations at three different locations of the Catalan coast. Strain ICMB1101 was isolated from Cubelles Beach (41°11′51.8″ N, 1°40′23.0″ E) in September 2019; strain ICMB1102 was isolated from Llavaneres Beach (41°33′07.5″ N, 2°29′31.6″ E) in July 2020; and strain ICMB1104 was isolated from La Fosca Beach (41°51′27″ N, 3°8′38″ E) in August 2020. Cell abundances of the host *Ostreopsis* sp. strain ICMB293 and the chytrid zoospores in each stock culture were determined. Culture flasks were inoculated in triplicate with healthy host cells and zoospores from each chytrid strain (ICMB1101, ICMB1102, ICMB1104), at a host:parasite ratio of 1:10 and a final volume of 20 mL. The initial abundances of *Ostreopsis* cells and zoospores were 300 mL^−^^1^ and 3000 mL^−^^1^, respectively. This low initial parasite abundance allowed to study the development of the infection from its early stage, and thus host-parasite dynamics to be monitored over time. The incubations were maintained at 21 °C and a 10 h:14 h light:dark cycle. The infections were followed for 20 days, with the flasks sampled on days 2, 5, 10, 16, and 20. The collected samples (2 mL) were fixed with formaldehyde (final concentration 3.6%) and then counted in a Sedgewick-Rafter chamber by light microscopy to determine the number of healthy and infected *Ostreopsis* cells as well as chytrid prevalence.

### Growth of chytrids using an alternative nutrient source

The ability of the chytrids to grow on organic sources other than dinoflagellate cells was examined using *D. arenysensis* strains ICMB1101, ICMB1103, and ICMB1104. Strain ICMB1103 is a monoclonal culture of *D. arenysensis* isolated from Tossa Beach (41°42′55.9″ N, 2°55′56.4″ E) in August 2020. Pine pollen (*Pinus pinea*) was added to the chytrid culture in a 10 mL 6-well plate. The presence of infections was checked by inverted light microscopy of the plates after 4 and 6 days of incubation. Thereafter, an inoculum of healthy *Ostreopsis* culture (strain ICMB293) was added to the wells to determine the viability of the sporangia and their ability to infect dinoflagellate cells.

## Results

### Diversity and relative abundance of the eukaryotic community

**Fig. 1:**
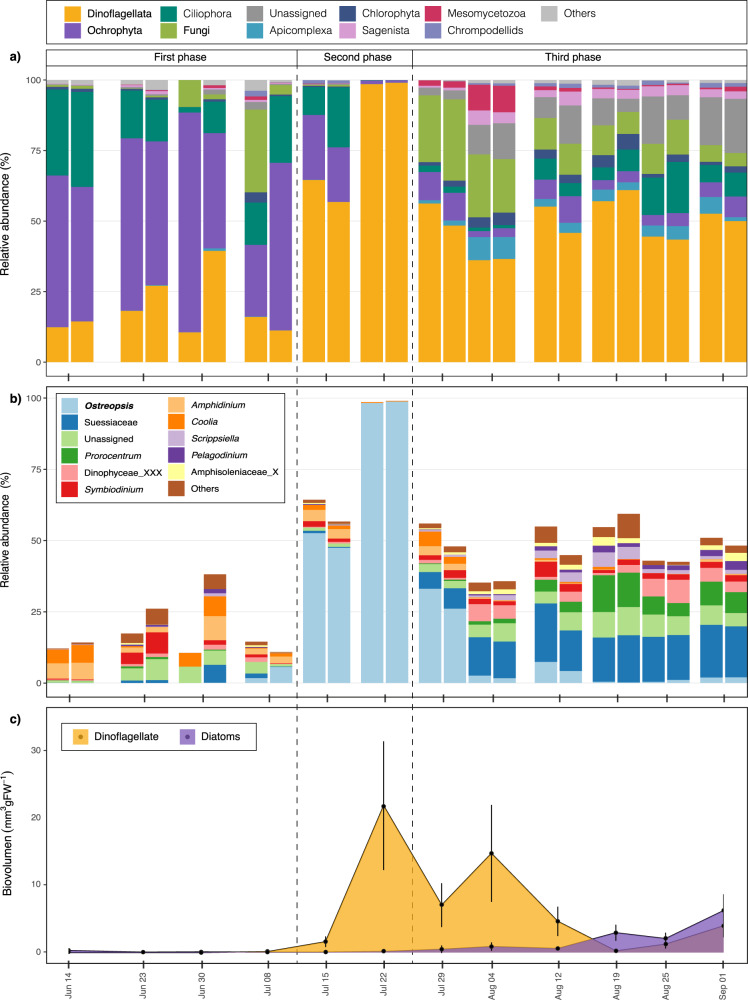
Temporal dynamics of the epiphytic assemblage on macroalgae. **a** Bar plot showing the variation (%), in replicates, of the different epiphytic eukaryote groups at each sampling date. **b** Bar plot showing the variation in the epiphytic dinoflagellate assemblage on macroalgae. Relative abundances (%) are based on the total protist community. **c** Non-stacked area plot showing the variations in the biovolumes of epiphytic diatom and dinoflagellate groups. Vertical dashed lines represent the three temporal phases described in the text.

The epiphytic protist community (8 to 100 μm fraction) on macroalgae was represented by 2153 ASVs, based on metabarcoding of the V4 18S rDNA region. The metabarcoding data could be categorized into three temporal phases according to community composition and the relative abundance of specific groups, including as Dinoflagellata, Ochrophyta (diatoms), Ciliophora, and Fungi (Fig. 1). The three phases of the community structure were observed from a Non-Metric Multidimensional Scaling (NMDS) analysis (Supplementary Fig. 1) and its statistical significance was confirmed through a Permutational multivariate analysis of variance using distance matrices (ADONIS. R^2^ = 0.48, *p*-value < 0.001).

The differentiation between theses phases was mainly attributed to the presence of distinct dominant taxonomic groups in each respective phase. The first phase, corresponding from June 14th to July 8th, was characterized by the dominance of Ochrophyta (diatoms), which accounted for > 50% of the relative abundance. This was followed by other groups of microalgae, including Dinoflagellata (dinoflagellates) and Ciliophora (Fig. 1a). In the second phase, observed from July 15th to July 22nd, the relative abundance of dinoflagellates ranged from > 60% to nearly 100%. During the third phase, from July 29th to September 1st, the relative abundance of dinoflagellates remained high (~ 50%) but that of other groups, including Fungi, Apicomplexa, Chlorophyta, Sagenista, Mesomycetozoa, and unassigned taxa, increased (Fig. 1a). However, microscopy of the samples showed slightly different dynamics of the epiphytic protist assemblage. The proportions of diatoms and dinoflagellates determined by microscopy were based on the biovolume (Fig. 1c). Thus, during the first phase, the biovolume of epiphytic diatoms and dinoflagellates was < 0.1 mm^3^ gFW^−^^1^. In the second phase, the fraction of epiphytic dinoflagellates increased to 21,8 mm^3^ gFW^−1^ while that of epiphytic diatoms remained unchanged. In the third phase, the dinoflagellate fraction reached a maximum of 14,7 mm^3^ gFW^−1^ (August 4th) followed by a progressive decrease to 0.2 mm^3^ gFW^−1^. During the month of August, the dominant fraction of organisms was dinoflagellates, consistent with the metabarcoding results. However, the biovolume of diatoms gradually increased such that by September the epiphyte assemblage was dominated equally by dinoflagellates and diatoms (Fig. 1c), which was not observed in the metabarcoding data.

### Diversity and relative abundance of the dinoflagellate assemblage

Given that dinoflagellates were the most abundant taxonomic group on macroalgae along the epiphytic succession, the structure of the epiphytic dinoflagellate assemblage was evaluated in detail. The metabarcoding results showed structural differences between the three phases described above (Fig. 1b), with a high diversity of species, including those belonging to the genera *Amphidinium, Coolia, Symbiodinium* and unassigned taxa, during the first phase. *Ostreopsis* sp. appeared in early July, with a relative abundance of < 6%. During the second phase, the relative abundance of this species increased from 40% to almost 100% of the dinoflagellate and protist sequences. During the third phase, the relative abundance of *Ostreopsis* sp. decreased from 25% (July 29th) to < 6% in subsequent samples. The composition of the dinoflagellate sequences during the third phase was more diverse than during the first and second phases and was dominated by Suessiaceae, unidentified taxa, and, to a lesser extent, *Prorocentrum* after August 4th (Fig. 1b). By contrast, according to the microscopic analyses *Ostreopsis* sp. dominated among the dinoflagellate during both the second and the third phases, accounting for 89% to almost 100% of the epiphytic dinoflagellate biomass (Fig. 1c). Other dinoflagellate species, belonging to the genera *Coolia*, *Amphidinium* and *Prorocentrum*, were detected at low abundance beginning on July 15th, consistent with the metabarcoding results.

### Diversity and relative abundance of fungal and chytrid assemblages

**Fig. 2:**
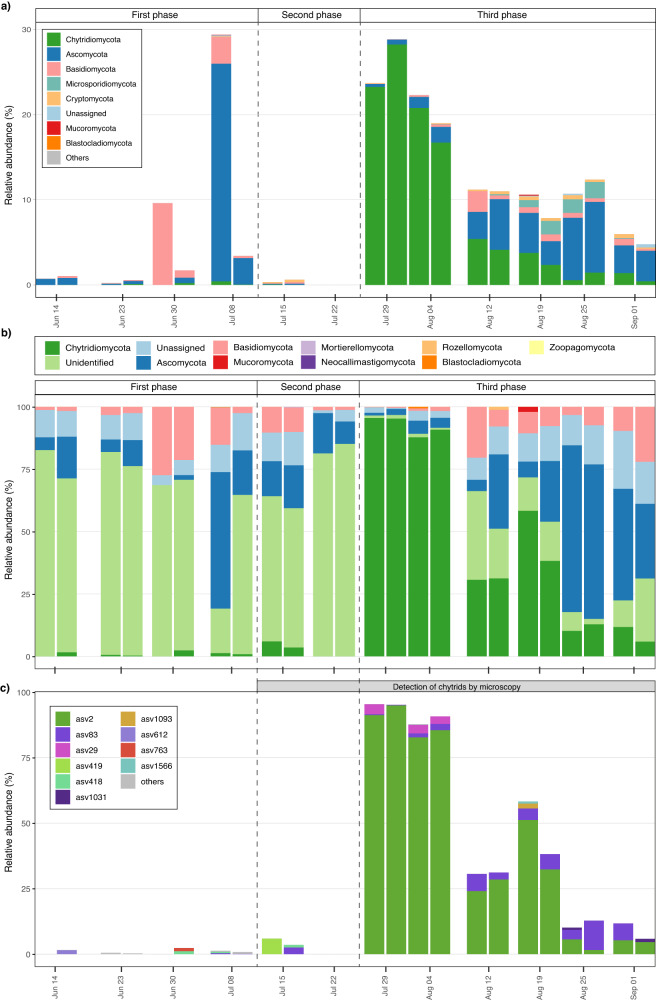
Temporal dynamics of the epiphytic fungal assemblage as determined using the V4 18S rDNA and ITS regions. **a** Bar plot showing the variation, in replicates, of the epiphytic fungal assemblage as determined using the V4 18S rDNA region. Relative abundances (%) are based on the total protist community. **b** Bar plot showing the relative abundance of the fungal groups as determined using the ITS region. **c** Bar plot showing the relative abundance of Chytridiomycota with respect to the fungal community based on the ITS region. The ten most abundant Chytridiomycota ASVs are shown; the remaining nine ASVs are included in the “other” category. The shaded area between (**b**) and (**c**) indicates the period when the presence of chytrids was detected by light microscopy, epifluorescence microscopy or incubation of natural samples.

The fungal community based on metabarcoding of the V4 18S rDNA region was represented by 203 ASVs. During the first and second phases, fungi accounted for < 1% of the protist community, with the exception of the samples taken on June 30th and July 8th, in which the inter-replicate values ranged from 2% to 30%. However, on July 29th, corresponding to the third phase, fungi accounted for almost 30% of the protist community, followed by a gradual decrease to < 5% by September 1st. (Fig. 1a). The analysis of the fungal assemblage revealed that the elevated relative abundances during the first phase were due to the contributions of Basidiomycota (June 30th) and Ascomycota (July 8th). During the third phase, Chytridiomycota emerged as the dominant group in the fungal community, but its contribution gradually declined and its dominance eventually alternated with that of Ascomycota (Fig. 2a). Between mid-August and September, overall fungal diversity increased.

The diversity of the fungal community was further examined using the ITS region of fungal representatives (Fig. 2b). Seventy-seven percent of the ITS sequences were discarded as they were not identified as belonging to fungi. A total of 466 ASVs were obtained, revealing differences in community composition during the three phases. During the first and second phases, the community was dominated by fungal sequences with unknown taxonomic affiliations, albeit with Ascomycota and Basidiomycota present to a lesser extent. Between July 29^th^ and August 4th, and thus during the third phase, the proportion of Chytridiomycota significantly increased and accounted for almost 100% of the fungal community. However, the relative abundance of Chytridiomycota gradually declined, accompanied by an increase in other groups, such as Ascomycota, Basidiomycota, and sequences of unclassified taxonomy.

Further analysis of the Chytridiomycota based on ITS region analysis revealed 19 ASVs (Fig. 2c). During the first and second phases, the composition of Chytridiomycota was diverse but the total relative abundance was < 5%. During the third phase, ASV2 emerged as the dominant Chytridiomycota sequence, accounting for almost 100% of the total Chytridiomycota and fungal sequences during the samplings of July 29th and August 4th, followed by ASV29 at much lower relative abundance. Although ASV2 remained the dominant Chytridiomycota sequence, its abundance relative to total fungal sequences gradually decreased. On August 25th and September 1st, the relative abundance of ASV2 was lower, and the relative abundance of ASV83 increased. ASV2 and ASV29 were only differentiated by one single nucleotide, showing an identity of 99.7%. Both sequences were taxonomically assigned to the species *Dinomyces arenysensis*, likely representing intraspecific variants. ASV83 was assigned as an unknown species of Lobulomycetales.

### Identification and prevalence of chytrids in field samples

**Fig. 3:**
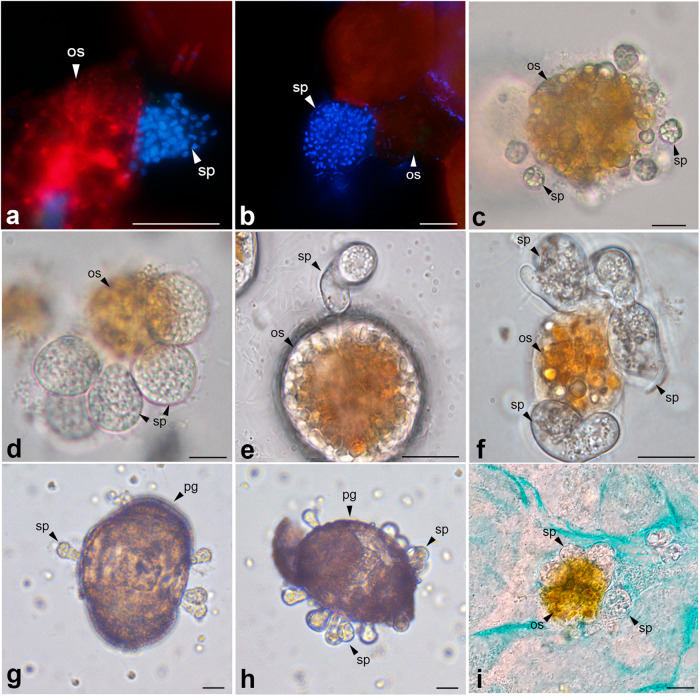
Microscopy images of chytrids. Epifluorescence microscopy: **a** DAPI staining of mature sporangia from natural samples. **b** DAPI staining of mature sporangia of *Dinomyces arenysensis* from laboratory cultures. Light microscopy. **c** Early infection stage of *D. arenysensis* strain ICMB1109 infecting *Ostreopsis* sp. (ICMB293). **d** Immature sporangium stage of strain ICMB1109 infecting *Ostreopsis* sp. **e** Early infection stage of Lobulomycetales strain ICMB1108 infecting *Ostreopsis* sp. **f** Immature sporangium stage of strain ICMB1108 infecting *Ostreopsis* sp. **g**, **h** Pine pollen grains with multiple sporangia of *D. arenysensis* ICMB1103, observed 4 days after inoculation of the chytrid (**i**) Alcian blue staining of mucilage present in cultures of Lobulomycetales sp. sp: sporangium, os: *Ostreopsis* sp., pg: Pine pollen grain. Scale bars = 10 µm.

Chytrid infections were detected by light and epifluorescence microscopy from the second phase (beginning July 15th) onwards (Fig. 3a, b and Table 1). During this period, two species of chytrids were identified by the molecular characterization of cultured strains established, or single cell isolates from natural samples (*Dinomyces arenysensis* and Lobulomycetales sp.). The last one was isolated, cultivated and identified as a new, yet undescribed species of Lobulomycetales (strain ICMB1108) (Fig. 3e, f and Table 1).

**Table 1:**
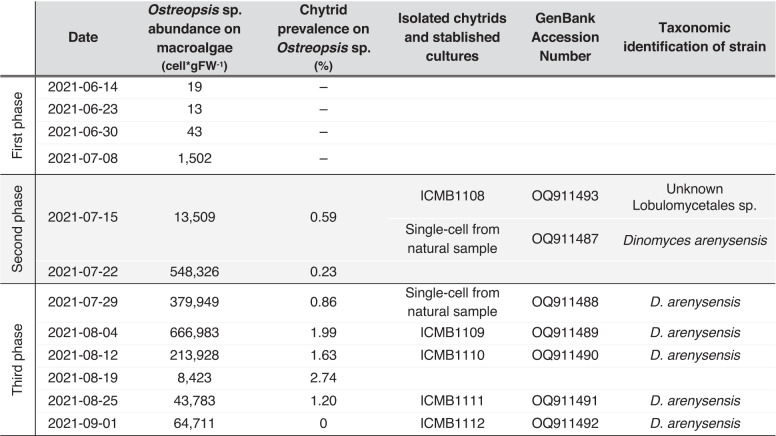
Sampling dates, cell abundance of *Ostreopsis* sp. on macroalgae as determined by light microscopy counts, and chytrid prevalence on *Ostreopsis* sp. in field samplings. –: not determined. Chytrids were isolated for culture and sequenced by single-cell PCR. Strains grown under laboratory conditions are named after the culture collection (ICMB). The 18S rDNA molecular sequences and the resulting taxonomic identities of the strains are indicated. White rows mark the first and third sampling-based phases of the epiphytic assemblage; shaded rows mark the second phase.

Only the prevalence of one species, *D. arenysensis*, was determined. The morphology of mature sporangia observed by epifluorescence was compared and confirmed with sporangia from cultures (Fig. 3a, b). The prevalence of *Dinomyces* infection during this second phase was < 0.5%. In the third phase, after August 4th, chytrid infections were recurrently detected in field samples by light microscopy, facilitating the establishment of chytrid cultures and thus their molecular characterization. Spherical epibiotic sporangia without discharge papillae were observed, and the subsequent molecular characterization of these strains revealed them to be *D. arenysensis* (ICMB1109–ICMB1112) (Fig. 3c, d and Table 1). Infection prevalence at this stage reached up to 2.7% (Table 1). Infections in the lab of the two chytrid species found in this study (*D. arenysensis* and Lobulomycetales sp.) were equally observed in the mucilage of *Ostreopsis* sp. (Fig. 3i). All infections, isolations and cultures were originated from the dinoflagellate *Ostreopsis* sp. No infections by chytrids were observed in any other microalgae during the study period.

### Facultative parasitic strategy of *D. arenysensis*

The ability of *D. arenysensis* to utilize non-algal organic matter for its life cycle and propagation was investigated by incubating previously established chytrid strains (ICMB1101, ICMB1103 and ICMB1104) with pine pollen grains. After 4 days of incubation, ICMB1104 was the only strain that had formed sporangia on the pollen grains, with chytrids growing in the main body of the grain and forming single or multiple sporangia (Fig. 3g, h). After 6 days, more mature sporangia were found, although no living *Ostreopsis* sp. cells from the initial inoculum were observed. The viability of the newly produced sporangia on the pollen was tested by inoculating new, healthy *Ostreopsis* sp. cells into the same well, which resulted in re-infection of the dinoflagellate by the chytrid 2 days later. These infections originated from zoospores released from the sporangia present on the pollen grains. Once the dinoflagellate cells were added and infected, no new infections on the pollen grains were observed.

### Temporal dynamics of *D. arenysensis* strains and *Ostreopsis* sp

**Fig. 4:**
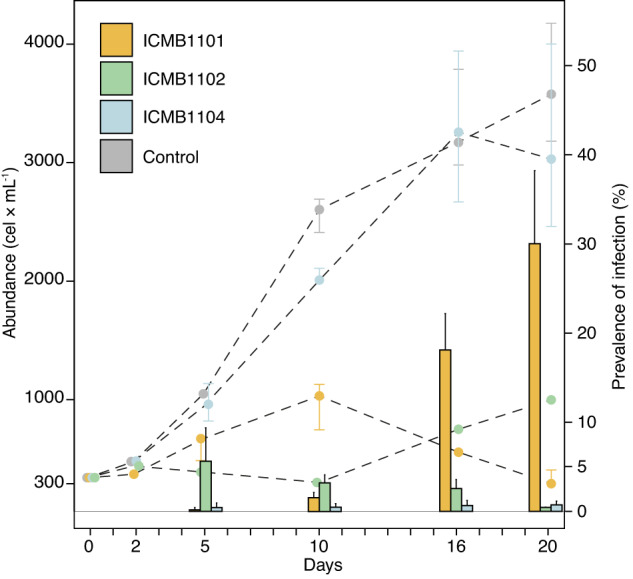
Temporal dynamics of infection. Cell abundance (cells mL^−1^) of host *Ostreopsis* sp. strain ICMB293 and the prevalence (%) of strains of the chytrid parasite *D. arenysensis* (ICMB1101, ICMB1102 and ICMB1104) over time (days). Dashed lines correspond to cell abundance, and bars to prevalence. Whiskers represent the standard deviation.

The levels of infection development and virulence differed among *Dinomyces* strains (ICMB1101, ICMB1102, ICMB1104) in infections of *Ostreopsis* sp. (strain ICMB293) (Fig. 4). In infections with strain ICMB1101, < 2% of *Ostreopsis* sp. cells were infected during the first 10 days of the experiment and the abundance of healthy *Ostreopsis* sp. cells gradually increased from 300 cells mL^−^^1^ to 900 cells mL^−1^. Thereafter, the prevalence of infection increased exponentially such that by the end of the experiment 30% of the population was infected and the population of healthy *Ostreopsis* sp. cells decreased to > 300 cells mL^−1^. When *Ostreopsis* sp. was inoculated with chytrid strain ICMB1104, the infection rate did not exceed 1% and the growth of *Ostreopsis* sp. cells was comparable to that observed in an uninfected control treatment. In infections with strain ICMB1102, the highest prevalence of infection occurred after 5 days, at which time 5% of the population was infected. On subsequent days, the prevalence decreased gradually, to < 1% on day 20. During the first 10 days of exposure, the *Ostreopsis* population remained stable, with cell numbers similar to the starting value. From day 10 until the end of the experiment, the *Ostreopsis* population increased from 300 cells mL^−1^ to 900 cells mL^−1^ while the prevalence of infection remained low.

## Discussion

This study investigated the diversity and temporal dynamics of marine epiphytic protist assemblages on macroalgae, with a focus on fungi, Chytridiomycota and dinoflagellates. Our findings provide new insights into the complex interactions between marine fungi and their host communities. Specifically, the results showed that both the composition and the relative abundances of fungal communities are influenced by the succession of epiphytic microalgae. Over time, the community structure of the epiphytic fungi changed, evidenced by changes in the dominance of Ascomycota, Chytridiomycota, and unidentified fungal organisms. The abundance of the Chytridiomycota was highly dependent on epiphytic dinoflagellate species assemblages, with *Dinomyces arenysensis* and a new species of Lobulomycetales as the primary representatives of this group. The peak in the abundance of the dinoflagellate *Ostreopsis* sp. coincided with the highest relative abundance of chytrids, particular *D. arenysensis*. Laboratory experiments showed that the latter species is characterized by intraspecific variability in its prevalence, with a capacity of growth on pollen demonstrated in one of the tested strains.

The identity and diversity of the eukaryotic protist community was explored using the V4 18S rDNA region, and that of the fungal community using the V4 18S rDNA and ITS regions. The two markers provide complementary information about the diversity of fungi present in the samples along with that of the other microbial components of the biofilm from which they were obtained. However, while V4 18S rDNA enables accurate assignments at high taxonomic levels, such as phylum and order, in fungal organisms the region is too conserved to allow species-level discrimination [49, 50]. The latter can be achieved with the ITS region, which allows precise taxonomic assignments at the genus and species levels. Nonetheless, the accuracy of the taxonomic assignments obtained with both markers heavily relies on the availability and quality of reference databases. The large number of unknown sequences in this study well demonstrates the need to expand our knowledge of fungal diversity. Identification of unknown fungal species will provide valuable insights into their ecological roles.

The main finding of the study is that the composition of the fungal community is influenced by the temporal dynamics of epiphytic protists on macroalgae. During the first phase, diatoms and dinoflagellates accounted for very few of the epiphytic protists on macroalgae. This phase can thus be considered as the initial period of colonization and development of the microalgae that will subsequently dominate the epiphytic community [22]. The fungal community on the macroalgae during the first phase was mainly composed of members of the Ascomycota and Basidiomycota, based on V4 18S rDNA. However, 70% of the fungal sequences obtained using the ITS region could not be attributed to any known fungal taxon, indicating a significant gap in our understanding of the diversity of marine fungal communities in these epiphytic assemblages, as has been reported in other studies [51–53]. As the protist biofilm on the macroalgae was not fully formed, this fungal assemblage may have been an epiphytic fungus of macroalgae rather than of the protist assemblage. In fact, Ascomycota and Basidiomycota are the main groups of marine fungi found in metabarcoding studies, and the most abundant and diverse fungal groups in benthic ecosystems [11, 54]. Studies conducted in the Mediterranean Sea have shown that these two groups, including Ascomycetes saprophytes of *Posidonia oceanica* and the macroalga *Dictyota dichotoma* [55], represent 97.2% of the fungal community present on vegetal substrates [56].

During the second phase, the abundance of epiphytic dinoflagellates increased rapidly, from < 25% to almost 100%. This proliferation corresponded specifically to the dinoflagellate *Ostreopsis* sp., a well-known blooming species characterised in previous studies of the NW Mediterranean during the summer months [22]. *Ostreopsis* sp. blooms are likely driven by the same factors that promote macroalgal growth, i.e., increases in temperature, irradiance, and substrate availability [57]. Furthermore, the mucilage secretions characteristic of these blooms enable the establishment of the microorganisms that will ultimately form the epiphytic summer biofilm on macroalgae [58]. The proportion of Ascomycota, Basidiomycota, and the unidentified set of taxa in the fungal community during the second phase remained the same as during the first phase. However, a week after the increase in *Ostreopsis* abundance, the fungal composition changed such that by the beginning of the third phase the fungal fraction accounted for nearly 25% of the total protist community, with an increase in the number of Chytridiomycota members, specifically *D*. *arenysensis*. The time lag between the maximum cell abundance of the host and the increase in parasite abundance aligns with typical predator-prey dynamics, where predator (in this case, parasite) and prey (host) alternate in abundance over time. Starting from a growing host population, the increase in this food resource will promote the growth of the parasite population. Parasitic pressure, in turn, can adversely affect host population abundance, leading to a subsequent decline in parasite population. The specific observed time lag can be considered as the period necessary for the parasite to complete its infective life cycle, in a situation of high host abundance [59]. In the case of chytrids, their complete development, from the onset of infection to the formation of new infective zoospores released into the environment, occurs within 2–3 days [10, 60]. A similar time lag was observed in studies that likewise evaluated the temporal dynamics of chytrids and their microalgal hosts [8, 61].

The abrupt increase in the relative abundance of Chytridiomycota during the beginning of the third phase coincided with the period of maximum infection prevalence in the samples and the highest abundance of *Ostreopsis* sp. This observation was consistent with the blooming strategy of chytrid species during phytoplankton blooms, which has been described as a ruderal strategy based on chytrid-phytoplankton parasitism during bloom events [62]. The subsequent decrease in dinoflagellate abundance occurred in parallel with an increase in other groups, such as Fungi, Apicomplexa, Mesomycetozoa, Sagenista and unassigned taxa. The dominant fungal group in this third phase was Chytridiomycota, although towards the end of this phase its abundance decreased and alternated with a relatively high abundance of Ascomycota. These changes, during the third phase, suggest that the decline of the dominant dinoflagellate *Ostreopsis*  sp. allowed the development of other protist groups, thereby changing the composition of the fungal community. This trend reflects changes in community assembly following the proliferation and decline of previously dominant *Ostreopsis* sp.

*Dinomyces arenysensis* was the dominant species in the fungal community examined in our study. This species is found in benthic marine-coastal habitats and has been described as a generalist, biotrophic parasite with a preference for the dinoflagellate *Ostreopsis* [9, 60]. However, the prevalence of *D. arenysensis* infections during the period of maximum *Ostreopsis*  sp. abundance was relatively low, ranging from 1% to 2.7%, which is comparable to the values reported for other host-parasite systems within plankton. For example, the prevalence of two chytrid species of the genus *Rhizosiphon* on the cyanobacterium *Anabaena* increased from non-detection to 6% [7]. In a study examining chytrids associated with freshwater diatoms, the prevalence of infection varied widely among chytrid species, with those exhibiting specialized parasitic behaviour attaining an infection prevalence as high as 70%. By contrast, the infection prevalence of species with facultative parasitic behaviour, and thus able to assimilate food resources other than diatoms, such as pollen, was low, around 2% [2]. The behaviour of *D. arenysensis* as observed in our study suggested that this species has a facultative parasitic life style. Previous research on the growth dynamics of *D. arenysensis* has reported its inability to grow on agar media or soil extracts, indicating an obligate parasitic lifestyle [60]. However, our laboratory experiments demonstrated that this chytrid can successfully use pine pollen, present in abundance in the atmospheric pollen above the Catalan Coast, to complete a viable life cycle. This ability to utilize diverse resources suggest that *D. arenysensis* is a facultative parasite. Moreover, it provides further evidence of intraspecies variability in virulence, as the ability to infect pine pollen was observed in only one of the studied strains. Likewise, sporangia were not observed on pollen when *Ostreopsis* sp. was present, indicating a preference for a parasitic life-style and that pollen serves only as a temporary resource. The ability of chytrids to infect both phytoplankton and pollen is common in freshwater environments [2, 63]. Our results suggest similar interactions in marine habitats, specifically in seasonal epiphytic assemblages, where other resources sustain the parasite until its preferred host again becomes available. However, survival of the parasite in winter, when the substrate of its host (macroalgae) is no longer available, suggests a reservoir within the plankton or benthonic compartment, allowing the recurrence of *Dinomyces* in *Ostreopsis* blooms [9].

Low infection prevalence of *Dinomyces* in *Ostreopsis* sp. could indicate benign parasitism (as described in ref. [64]). This suggests that chytrids and dinoflagellates can coexist without significant harm to the host population, allowing for a recovery from parasite pressure. Previous studies suggested that marine chytrid outbreaks prematurely terminate or suppress phytoplankton blooms [65, 66]. However, this was not supported by the prevalence of infection observed in the field in our study, as the infections observed in natural samples did not explain the decrease in *Ostreopsis* abundance. Rather, our findings are consistent with the coexistence of chytrids and dinoflagellates. In fact, laboratory studies have shown that parasites and dinoflagellate host populations can coexist if the equilibrium between them is sustained by a minimum host density threshold [67]. Coexistence at low infection prevalence was also observed in studies of other protist parasites, such as *Amoebophrya* sp., and can be interpreted as reducing the fitness of the dinoflagellate host without causing significant damage to the host population [68].

In contrast to the field studies suggesting coexistence, in the lab experiments the impact of parasitism ranged from harmless to lethal, depending on the host affinity of the different strains of *D. arenysensis*. Thus, the coexistence between chytrids and dinoflagellates observed in the field might reflect differences in the virulence of the different strains of chytrids. The presence in the field of multiple parasite strains with varying degrees of virulence would support parasite-host equilibrium and thus coexistence. Studies of the relationship between chytrids and cyanobacteria have shown that differences in the performance of multiple strains of parasitic chytrids can lead to changes in the infection strategy of these organisms [69]. Intraspecific diversity can also promote species coexistence and influence community dynamics, by promoting niche complementarity between genotypes [70, 71].

Mucilage secretion, a prominent feature of *Ostreopsis* and another microalgal species, provides structure to the epiphytic assemblage [72, 73], and serves as a trap for prey acquisition [74]. In a recent study, it has been proposed that the mucilage acts as a protective barrier against chytrid infection in diatoms [75]. However, the two chytrid species identified in our study (*D. arenysensis* and Lobulomycetales sp.) were capable of infecting the dinoflagellate *Ostreopsis* despite mucilage secretion. The ability to overcome the protective mucilage barrier suggests that these chytrid species have evolved specific mechanisms to infect their host. Understanding these mechanisms could provide valuable insights into the interactions between fungi and dinoflagellates, and the competitive advantage that means using these resources.

In summary, this study characterized epiphytic protist succession on macroalgae with focus on chytrids and dinoflagellates. Our investigation of the host-parasite dynamics of *Dinomyces arenysensis* showed that the infection peak is influenced by the predominant resource, *Ostreopsis* sp., but also that the parasite is facultative with respect to its host resources. The coexistence of chytrids and dinoflagellates without significant harm to the host organism was supported by field studies. Concurrently, laboratory experiments indicated that this coexistence is partly attributed to the variability in the virulence levels exhibited by different strains of chytrids. Our findings contribute to a better understanding of the dynamics of epiphytic biofilm and host-parasite interactions in less-explored marine benthic habitats.

### Supplementary Information

Supplementary Figure 1

Supplementary Dataset 1

## Data Availability

The datasets presented in this study are available in the NCBI Sequence Read Archive under Bioproject numbers PRJNA891539, PRJNA891506, and are included in the supplementary datasets of this published article.
