# Supplementary material for: The succession of epiphytic microalgae conditions fungal community composition: how chytrids respond to blooms of dinoflagellates
Source: ISME Commun. 2023 Sep 26;3:103. doi: 10.1038/s43705-023-00304-x (PMC10522651; doi:10.1038/s43705-023-00304-x)

SUPPORTING INFORMATION

Supplementary Figure 1. Non-metric multidimensional scaling (NMDS) conducted using samples obtained during the three temporal sampling phases.

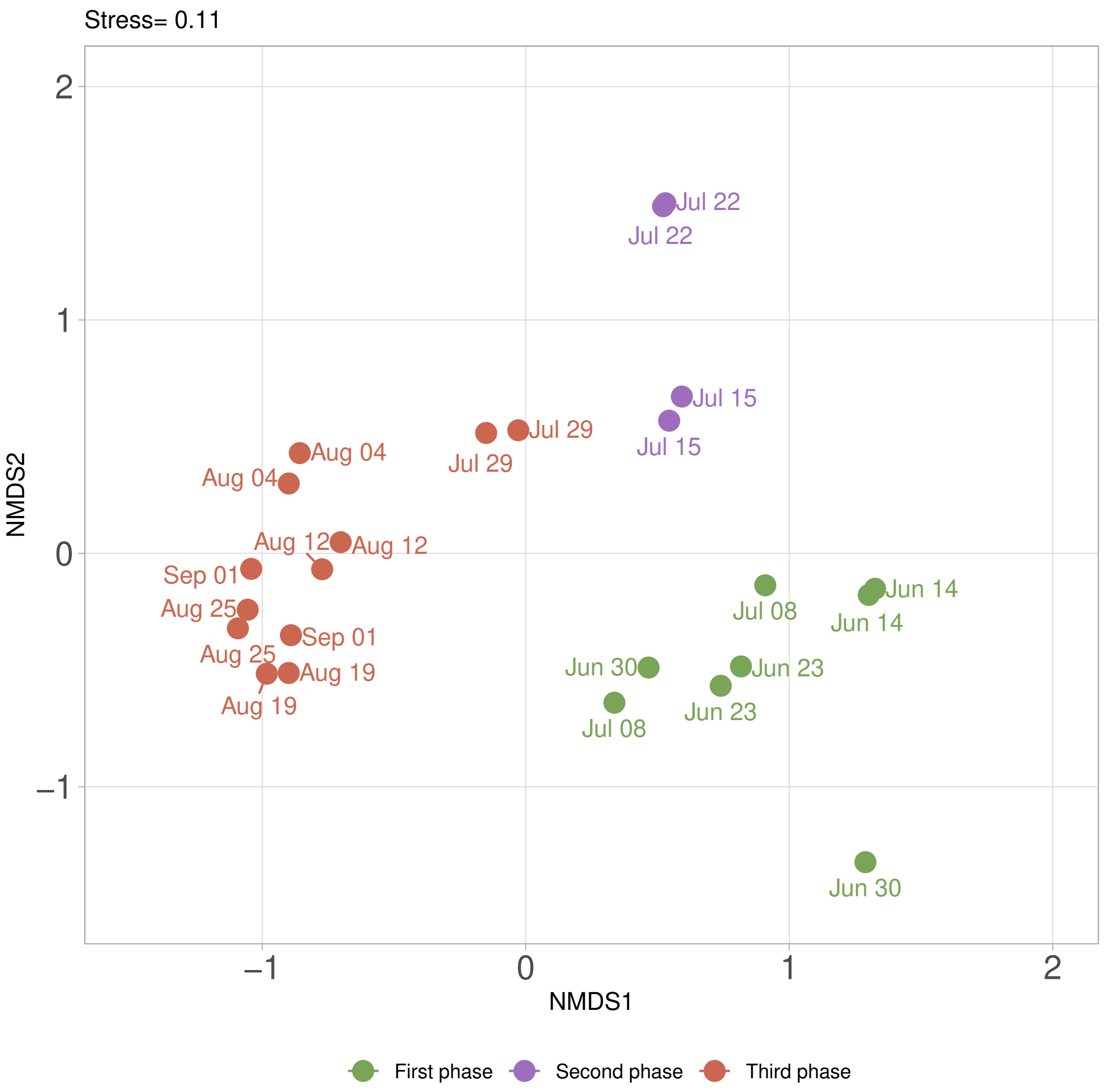

Supplement: Supplementary file 1 — Supplementary Figure 1 [file 43705_2023_304_MOESM1_ESM.pdf]
